# Effectiveness of Recombinant Human Growth Hormone Therapy for Children With Phelan-McDermid Syndrome: An Open-Label, Cross-Over, Preliminary Study

**DOI:** 10.3389/fpsyt.2022.763565

**Published:** 2022-02-16

**Authors:** TianXiao Li, Ruijin Xie, Jinling Zhao, Hua Xu, Ying Cui, Chenyu Sun, Chunhong Wang, Yueying Liu

**Affiliations:** ^1^Affiliated Hospital of JiangNan University, Wuxi, China; ^2^Wuxi School of Medicine, Jiangnan University, Wuxi, China; ^3^AMITA Health Saint Joseph Hospital Chicago, Chicago, IL, United States

**Keywords:** Phelan-McDermid syndrome, recombinant human growth hormone, insulin-like growth factor-1, cross-over, open-label

## Abstract

**Background:**

Phelan-McDermid syndrome (PMS), also known as the 22q13. 3 deletion syndrome, is a rare neurodevelopmental syndrome with approximately 2,800 patients reported worldwide. Previous pilot study demonstrated that IGF-1 could significantly improve in both social impairment and restrictive behaviors of the patients. However, most of the patients in the developing countries like China cannot afford the high cost of using IGF-1. Our research team speculated that rhGH might serve as a low-cost and more accessible treatment for PMS. Therefore, the purpose of this open-label, cross-over, pilot study was to further investigate the safety and efficiency of rhGH in patients with PMS.

**Methods:**

A total of six children with PMS were enrolled in in this open-label, cross-over, pilot study. The children were randomly divided into two different groups. Group A received placebo followed by rhGH, while group B was treated with rhGH first. Neuropsychological and behavior assessments of the patients were performed before the stage I of study and 3 months after the intervention of stage I. After a 4-week period of washout, these assessments were conducted again before the stage II of study and 3 months after the intervention of stage II. Serum insulin-like growth factor-1 (IGF-1) and insulin-like growth factor binding-protein (IGFBP)-3 were also evaluated monthly during the intervention phases of the pilot study

**Results:**

Compared with the placebo, rhGH treatment significantly decreased subscale scores of GDS (*P* < 0.0085) and trended to improve the total scores of GDS (*P* < 0.05), while the total scores and subscale scores of SC-ABC significantly decreased (*P* < 0.0085) following 3-months rhGH treatment. The similar results were also observed in comparison with baseline. Compared with the baseline, the level of serum IGF-1 and IGFBP-3 increased significantly (*P* < 0.05) following 3-months rhGH treatment, while the placebo group had no significant impact on serum IGF-1 and IGFBP-3 (*P* > 0.05). One child developed skin allergy the day after the first rhGH treatment, which were resolved later.

**Conclusions:**

In summary, this pilot study involving six PMS children patients reveals that rhGH has a positive treatment effect on PMS. These results encourage the undertaking of a large, randomized placebo-controlled trial to conclusively prove rhGH efficacy and tolerability in PMS, thereby promoting it as a low-cost, more accessible treatment for PMS, as compared to IGF-1.

## Introduction

Phelan-McDermid syndrome (PMS), also known as the 22q13.3 deletion syndrome, is a rare neurodevelopmental syndrome with approximately 2,800 patients reported worldwide (1). PMS is often characterized by seizures, global developmental delay, intellectual disability, severe speech delay, hypotonia, and autism spectrum disorder (ASD) (2, 3). In addition, these children often have minor dysmorphic facial features such as plump flesh, rounded face, long eyelashes, pointed chin, prominent/dysplastic ears, bulbous nose, full lips, dysplastic nails, and dolichocephaly (4). Most of the patients with PMS have terminal chromosomal deletions with deleting sizes ranging from hundreds of kilobases (kb) to over nine megabases (Mb) in the 22q13 region (5). Previous genotype-phenotype studies of PMS implied that PMS can also be caused by: (1) interstitial chromosomal deletions; (2) unbalanced translocations or other chromosomal rearrangements, resulting in ring chromosomes; (3) point mutations in the *SHANK3* gene (6). Almost all the patients with PMS are associated with SH3 and multiple ankyrin repeat domains 3 (*SHANK3*) gene malfunction/deficiency. Meanwhile, loss or inactivation of one *SHANK3* allele is sufficient to be diagnosed as PMS (3, 6). Interestingly, although the genotype–phenotype correlations in PMS are complex, recent advanced genotype-phenotype analyses have shown that dysmorphisms, comorbidities, speech status, and ASD may correlate to the deletion size of *SHANK3* gene (7–9).

*SHANK3* is a key gene, encoding a scaffolding protein enriched in the postsynaptic density of glutamatergic synapses, which plays a critical role in synaptic development and maturation (3, 10). Reduced expression of *SHANK3* gene will cause the loss of dendrites and the dysfunction of synaptic transmission and plasticity (3). In addition, point mutations of the *SHANK3* gene account for ~1% of the idiopathic forms of autism spectrum disorder, and *SHANK3* haploinsufficiency is responsible for the major neurological features of PMS (10).

Although nearly 40 years have been passed since the first report of PMS in 1985 (11), there is still a long way to go for exploring the effective treatment for PMS. Currently, the most mentioned and recommended medication for PMS treatment is insulin-like growth factor-1 (IGF-1). IGF-1 is a neurotrophic factor possessing growth hormone-like activities. It can cross the blood-brain barrier and improve synapse development by promoting neuronal cell survival, synaptic maturation, and synaptic plasticity (12, 13). Previous study has shown that IGF-1 could reverse the electrophysiological deficits observed in *Shank3*-deficient mice and the induced pluripotent stem cells (iPSCs) from patients with PMS, indicating that IGF-1 could strengthen excitatory synaptic transmission and reduce the rate of NMDA-receptors decay (3, 12, 14). In addition, a cross-over, randomized, pilot study (NCT01525901) including nine 5- to 15-year-old children with PMS demonstrated that IGF-1 could significantly improve in both social impairment and restrictive behaviors of the patients (15). However, most of the patients in the developing countries like China cannot afford the high cost of using IGF-1. Therefore, it is necessary to develop a cheaper and efficient alternative treatment.

Recently, our research team published a case report, in which a Chinese child with PMS improved his motor skills and autism-like behaviors after the treatment of recombinant human growth hormone (rhGH) (16). Based on this, we speculated that rhGH might serve as a low-cost and more accessible treatment for PMS. Therefore, the purpose of this cross-over, placebo-controlled, randomized, pilot study was to further investigate the safety and efficiency of rhGH in patients with PMS, focusing on neurodevelopmental and behavior measurements.

## Materials and Methods

### Participants

A total of six children (four boys and two girls) with PMS enrolled in this open-label, cross-over, pilot study through email and telephone messages (Table 1). The age of the subjects ranged from 1 to 5 years old [mean = 3.00, standard deviation (SD) = 1.90]. The chromosomal deletion size (Mb), detailed clinical symptoms, and other information of these children shown on Table 2. The diagnosis of ASD was based on the Diagnostic and Statistical Manual of Mental Disorders, the 5th Edition (DSM-5) or the Screening Tool for Autism in Toddlers and Young Children (STAT) for early diagnosis (17). Exclusion criteria included active or suspected tumor, intracranial hypertension, chronic kidney disease, acute proliferative or severe non-proliferative diabetic retinopathy, allergy to rhGH or severe comorbidity.

**Table 1:** General clinical data of subjects.

**Subject**	**Gender**	**Age (month)**	**SC-ABC**	**DQ**
1	Girl	18.4	61	59.6
2	Boy	60.3	79	35
3	Boy	31.7	58	61
4	Boy	36.5	76	59
5	Girl	48.6	68	68
6	Boy	54.3	81	54

*SC-ABC, Simplified Chinese version of the Aberrant Behavior Checklist; DQ, development quotient*.

**Table 2:** Details of the 22q13.3 deletions identified in 6 individuals with PMS.

**ID**	**Evaluation method**	**Rearrangement**	**Array coordinates (hg19)**	**Deletion size (Mb)**	**Genes**	**Clinical symptoms**	**Dysmorphic features**
1	aCGH	Deletion	Chr22:51159318-51159319	0.022	*SHANK3*	Global developmental delay; intellectual disability, impaired adaptive behavior, severe delayed speech intellectual disability. autism-like behaviors	Large fleshy hands, rounded face
2	MLPA	Deletion	Chromosome 9-22	NA	Many (From *ABCA4* to *SPTLC1)*	Global developmental delay, intellectual disability, impaired social behavior, absent of speech and language, combined with epilepsy	Prominent ears, pointed chin
3	aCGH	Deletion	Chr22:46352183-51244174	4.891	Many (From *CPT1B* to *SHANK3)*	Autism-like behaviors: global developmental delay; intellectual disability, impaired adaptive behavior, absent speech	Large fleshy hands, rounded face
4	aCGH	Deletion	Chr22:50155448-51197766	1.029	Many (From *BRD1* to *ACR)*	Autism-like behaviors: developmental regression, global developmental delay intellectual disability, impaired social behavior, severe speech and language, impulsive and grumpy	Pointed chin, full eyelids
5	aCGH	Deletion	Chr22:47264176-51206348	3.9	Many (From *ACR* to ZEBD4*)*	Global developmental delay intellectual disability, impaired social behavior, absent speech and language, stereotyped action	Large fleshy hands, rounded face
6	aCGH	Deletion	Chr22:46594261-51220752	5.8	Many (From *ACR* to ZEBD4	Global developmental delay intellectual disability, impaired social behavior, absent speech and language, reduced perception of pain, impulsivity	Large fleshy hands, rounded face, prominent ears

*NA, not available*.

### Study Design

**Figure 1:**
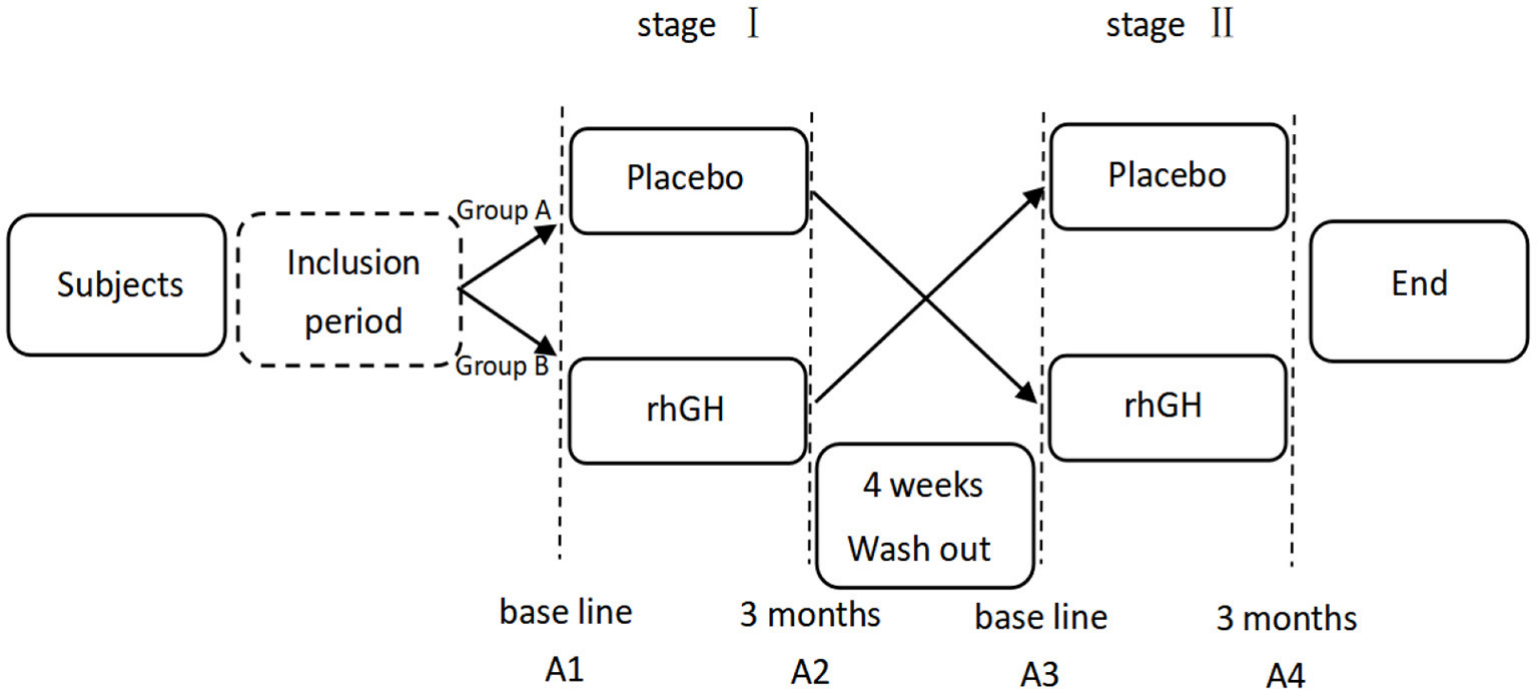
Flow chart of the study protocol. A1/A2-A3/A4 stands for monitoring the treatment effect before and 3 months after the intervention of each stage.

This study was an open-label, cross-over, pilot study (Figure 1). Subjects were randomly divided into two different groups. Group A received placebo followed by rhGH, while group B was treated with rhGH first. Randomization of the treatment (i.e., starting with rhGH or placebo) was performed through a permuted four-block designed by the physician, who was the only person not blinded for the treatment allocation. We established a 4-week washout period between the treatment stages based on the existing clinical trials on the pharmacokinetics of rhGH and IGF-1 (15, 16), which is sufficiently long for active rhGH metabolites removal according to their mean half-lives (~30 min). Following the washout period, the children received the other treatment. Each treatment stage (rhGH and placebo) lasted for 3 months.

### Ethical Approval

The parents of the children participating in this pilot study were informed about the potential adverse reactions and provided written informed consent. The Research Ethics Committees of the Affiliated Hospital of JiangNan University (WuXi, China) approved this study (No. LS2020017). The study was also registered in the ClinicalTrials.gov Protocol Registration System, registration number NCT05105685.

### Drug Interventions

Placebo and rhGH (Changchun Jinsai Pharmaceutical Co., Ltd, S20080011) were used for interventions in this pilot study. The placebo was normal saline from the same package and was injected at the same dose as rhGH. The rhGH treatment or placebo was started at 0.1 IU/kg once daily based on both the official Chinese guidelines for the use of rhGH and our previous study (16, 18). The injection sites included the outer mid-section of thigh, the upper arm, and the abdominal wall, while injections at the same site within a 1-month interval were not allowed. The distance between successive injections was longer than 1.0 cm to prevent subcutaneous tissue degeneration and potentially adverse effects of rhGH.

### Main Measurements

#### Neuropsychological and Behavior Measurements

Neuropsychological and behavior assessments of the patients were performed before the stage I of study and 3 months after the intervention of stage I. After a 4-week period of washout, these assessments were conducted again before the stage II of study and 3 months after the intervention of stage II (Figure 1). The neuropsychological development was measured by using Chinese version of the Gesell Development Scale (GDS), which was revised by the Beijing Children's Health Care Unit in 1986. The GDS consists of four subscales, evaluating the motor development, language development, adaptive skills, and personal social behavior of children. Motor development is divided into gross motor function and fine motor function, which can measure the neural development of children between 0 and 5 years old with ASD. The development quotient (DQ) in the GDS was used to quantify neurodevelopment, which can indicate the level of neurodevelopment and is interpreted as follows: DQ = 86 as normal, DQ 76–85 as marginally delayed, DQ at 55–75 as slightly delayed, DQ at 40–54 as moderately delayed, and DQ ≤ 39 as severely delayed.

The children were also evaluated based on the simplified Chinese version of the Aberrant Behavior Checklist (SC-ABC) (Supplementary File 1). Different from its English version that created by Krug in 1980 (19), SC-ABC was based on the study of Krug in 2009, in which proved ABC can be used in 14 months children (20), then it was translated into Simplified Chinese by the researchers of Peking University Sixth Hospital. To date, the SC-ABC scale has been verified and widely used for more than 10 years to assess the changes of behavioral symptoms in Chinese children aged from 14 months to 14 years old with ASD (21, 22). This checklist includes 57 items and five subscales: sensory behavior, social relating, body and object use, language and communication skills, and social and adaptive skills. Each item was scored from 0 to 3, with higher scores indicate more severe symptoms.

#### Biochemical Measurements

IGF-1 and insulin-like growth factor binding protein 3 (IGFBP-3) were evaluated monthly during the intervention phases of the pilot study. All the laboratory indices were recorded in the morning while the children fasted *via* chemiluminescent immunometric assay (CLIA) method and analyze in the laboratory of our hospital (23, 24).

### Adverse Events

AEs were measured during the study via monitoring visits or phone calls through an adapted semi-structured interview every 2 weeks based on the literature (25) (Supplementary File 2) and extensive clinical evaluations every 4 weeks, which were recorded by investigators or research staff members. The AEs were documented for severity, duration, and management.

### Statistical Analyses

All statistical analyses were performed using SPSS 23.0 software (IBM Corporation, Armonk). Data were reported as mean ± SD. Adverse events results were reported as numbers and percentages. ANOVA was implemented to compared the different effects of the orders of giving placebo or rhGH first, and to compare whether differences of GDS and SC-ABC exits at baseline. Following completion of the treatment, independent sample *t*-tests were conducted to compare main measurements of two groups. Paired *t*-test was conducted to compare main measurements between the baseline and after 3 months intervention. Bonferroni's correction was conducted to control for the probability of committing a type I error when multiple comparisons of the scores of the GDS and SC-ABC or the levels of serum IGF-1, IGFBP-3, P_new_ = P_original_/*n* (*n*: The total number of comparisons or tests being performed) (26–28). The trend threshold is set at *P* < 0.05 and corrected significance threshold is set at *P* < 0.0083 (*n* = 6) in these test.

## Results

### Effect of Orders

The results were shown in the Table 3. The sequence of administration had no significant effect on treatment efficacy, and no statistically significant differences were found on the serum level of IGF-1 (*F* = 0.156, *P* = 0.071), IGFBP-3 (*F* = 0.687, *P* = 0.454), total scores of GDS (*F* = 0.643, *P* = 0.468), and SC-ABC (*F* = 0.007, *P* = 0.938) in different orders. Meanwhile, we found significant differences on the main measurements after rhGH treatment (*P* < 0.0083), which means rhGH treatment can take effect on the main measurements.

**Table 3:** Analysis of variance of crossover data on main measurements value in two groups.

**Source of variation**	**DQ**		**SC-ABC**		**IGF-1**		**IGFBP-3**	
	***F***	***Nominal P***	***F***	***Nominal P***	***F***	***Nominal P***	***F***	***Nominal P***
Order	0.643	0.468	0.007	0.938	0.156	0.071	0.687	0.454
Subject	86.190	**0.000**	11.828	**0.016**	8.171	**0.032**	2.807	**0.002**
Treatment	225.804	**0.000**	192.980	**0.000**	85.951	**0.001**	6.124	**0.048**

*SC-ABC, Simplified Chinese version of the Aberrant Behavior Checklist; DQ, development quotient; IGFBP-3, insulin-like growth factor binding protein 3; IGF-1, insulin-like growth*

*factor-1*.

*Nominal P-value is set at 0.05, corrected statistical significance is set at 0.0083. Values that were lower than 0.0083 would be shown in bold*.

### Changes in GDS

We compared the total scores and subscale scores of GDS to measure the neuropsychological changes between the baseline and following 3-months of rhGH treatment in both groups (Table 4). When compared with the placebo, rhGH treatment trended to improve the total scores of GDS (*P* < 0.05). When compared with the baseline, the DQ score also improved (*P* < 0.0083). Furthermore, significant improvements were observed in the subscale scores of GDS after 3 months of treatment (Table 5), in comparison with those of the baseline. These changes included: gross motor function (*P* < 0.0083), fine motor function (*P* < 0.0083), language development (*P* < 0.0083), adaptive skills (*P* < 0.0083), and personal social behavior (*P* < 0.0083). The effect sizes of the rhGH treatment by measuring the total scores difference of the GDS between two groups were also provided (*r* = 0.549).

**Table 4:** Comparison of the total scores of the GDS before and after treatment.

**Projects**	**Placebo (*****n*** **= 6)**		**rhGH (*****n*** **= 6)**		***t***	***Nominal P-value***	**Cohen's *d***	**Effect size**
	**Mean**	**SD**	**Mean**	**SD**				
Baseline	57.47	11.89	56.60	11.10	0.131	0.999		
3 months	58.31	12.75	76.11	14.29	−2.277	0.046	1.314	0.549
*t*	−2.103	−9.562	002F	/	/	/		
Nominal P-value	0.089	**<0.001**	/	/	/	/		

*GDS, Gesell Development Scale*.

*Nominal P-value is set at 0.05, corrected statistical significance is set at 0.0083. Values that were lower than 0.0083 would be shown in bold*.

**Table 5:** Comparison of the subscale scores of the GDS before and after treatment.

**Projects**	**Baseline (*****n*** **= 6)**		**3 months (*****n*** **= 6)**		***t***	***Nominal P*-value**
	**Mean**	**SD**	**Mean**	**SD**		
**Gross motor**						
Placebo	53.45	22.57	54.00	22.27	−1.464	0.203
rhGH	52.50	22.15	79.27	16.87	−7.309	**0.001**
**Fine motor**						
Placebo	51.62	21.40	53.05	22.46	−1.609	0.169
rhGH	50.00	20.59	73.85	25.10	−8.529	**<0.001**
**Language**						
Placebo	48.57	22.54	48.88	22.02	−0.583	0.585
rhGH	47.67	21.92	68.82	28.60	−6.556	**0.001**
**Adaptive skills**						
Placebo	65.33	8.51	66.17	9.61	−1.185	0.289
rhGH	65.17	8.54	82.07	6.59	−8.372	**<0.001**
**Personal social behavior**						
Placebo	63.88	8.10	64.82	8.09	−1.444	0.208
rhGH	63.17	9.50	76.50	9.68	−4.050	**0.010**

*GDS, Gesell Development Scale*.

*Nominal P-value is set at 0.05, corrected statistical significance is set at 0.0083. Values that were lower than 0.0083 would be shown in bold*.

### Changes in SC-ABC

We compared the total scores and subscale scores of SC-ABC to measure the behavior changes between baseline and following 3-months rhGH treatment in both groups (Table 6). When compared with the placebo, rhGH treatment significantly decreased the total scores of SC-ABC (*P* < 0.0083). When compared with the baseline, the total score (*P* < 0.0083). The similar results were also observed in the subscale scores of SC-ABC after 3 months of treatment, in comparison with baseline (Table 7), including sensory behavior (*P* < 0.0083), social relating (*P* < 0.0083), body and object use (*P* < 0.0083), language and communication skills (*P* < 0.0083), and social and adaptive skills (*P* < 0.0083). The effect sizes of the rhGH treatment by measuring the total scores difference of the SC-ABC between two groups were also provided (*r* = 0.847).

**Table 6:** Comparison of total SC-ABC score before and after treatment.

**Projects**	**Placebo (*****n*** **= 6)**		**rhGH (*****n*** **= 6)**		***t***	***Nominal P*-value**	**Cohen's *d***	**Effect size**
	**Mean**	**SD**	**Mean**	**SD**				
Baseline	70.17	9.45	70.50	10.01	0.088	0.693		
3 months	70.00	9.01	41.83	8.64	5.528	**<0.001**	3.191	0.847
*t*	1.397	13.717	/	/	/	/		
Nominal P-value	0.221	**<0.001**	/	/	/	/		

*SC-ABC, Simplified Chinese version of the Aberrant Behavior Checklist*.

*Nominal P-value is set at 0.05, corrected statistical significance is set at 0.0083. Values that were lower than 0.0083 would be shown in bold*.

**Table 7:** Comparison of subscale scores of SC-ABC before and after treatment.

**Projects**	**Baseline (*****n*** **= 6)**		**3 months (*****n*** **= 6)**		***t***	***Nominal P*-value**
	**Mean**	**SD**	**Mean**	**SD**		
**Sensory behavior**						
Placebo	11.50	3.02	11.33	2.98	1.000	0.363
rhGH	11.67	2.88	7.33	1.97	5.398	**0.003**
**Social relating**						
Placebo	17.00	2.28	16.50	2.26	1.000	0.363
rhGH	16.83	2.14	10.17	2.56	10.847	**<0.001**
**Body and object use**						
Placebo	17.67	2.80	18.17	2.71	−1.464	0.203
rhGH	17.50	2.74	10.67	2.80	4.722	**0.005**
**Language and communication skills**						
Placebo	12.67	4.55	12.00	4.05	2.000	0.102
rhGH	13.00	5.22	7.17	2.71	4.238	**0.008**
**Social and adaptive skills**						
Placebo	11.67	1.51	11.83	2.04	−0.255	0.809
rhGH	11.67	1.51	6.50	0.84	7.900	**0.001**

*SC-ABC, Simplified Chinese version of the Aberrant Behavior Checklist*.

*Nominal P-value is set at 0.05, corrected statistical significance is set at 0.0083. Values that were lower than 0.0083 would be shown in bold*.

### Changes in Biochemical Measurements

Compared with the baseline, the level of serum IGF-1 and IGFBP-3 trend to increased (*P* < 0.05) following 3-months rhGH treatment, while the placebo group had no significant impact on serum IGF-1 and IGFBP-3 (*P* > 0.05) (see Table 8). The effect size of the changes in the level of serum IGF-1 and IGFBP-3 is *r* = 0.736 and *r* = 0.623, respectively, which in turn prove rhGH can take effect *via* increase the level of serum IGF-1 in circulation.

**Table 8:** Comparison of IGF-1, IGFBP-3 before and after treatment.

**Projects**	**Placebo (*****n*** **= 6)**		**rhGH (*****n*** **= 6)**		***t***	***Nominal P*-value**	**Cohen's *d***	**Effect size**
	**Mean**	**SD**	**Mean**	**SD**				
**IGF-1**								
Baseline	122.41	24.96	122.35	24.36	0.004	0.974		
3 months	123.97	24.43	254.73	81.38	−3.770	**0.004**	2.176	0.736
*t*	−1.646	−4.657	/	/	/	/		
Nominal *P*-value	0.161	0.006	/	/	/	/		
**IGFBP-3**								
Baseline	3.43	0.60	3.49	0.63	−0.170	0.948		
3 months	3.42	0.72	4.60	0.76	−2.766	0.020	1.594	0.623
*t*	0.006	−3.081	/	/	/	/		
Nominal *P*-value	0.950	0.027	/	/	/	/		

*IGFBP-3, insulin-like growth factor binding protein 3; IGF-1, insulin-like growth factor-1*.

*Nominal P-value is set at 0.05, corrected statistical significance is set at 0.0083. Values that were lower than 0.0083 would be shown in bold*.

### Adverse Events Results

We found that rhGH was well tolerated by the patients during treatment, with no serious AEs observed. One child developed skin allergy the day after the first rhGH treatment, with red plaques on the chest, back, legs, and face. After 2 days of treatment with dry desloratadine suspension, the symptoms were resolved. He did not exhibit additional allergic signs after continuous further use of rhGH. During the study period, all the children tolerated the dose of rhGH treatment.

## Discussion

To the best of our knowledge, this is the first reported pilot study aiming to investigate the safety and efficiency of rhGH in patients with PMS. Growth hormone (GH) is a 191-amino acid, single-chain polypeptide, which is secreted by pituitary gland (29). GH has well-established roles in stimulating cell growth, reproduction, and regeneration (30). IGF-1 is a 70-amino acid polypeptide hormone primarily produced in the liver, which is well known to promote the peripheral effects of GH and serve as the mediator factor for GH's actions. IGF-1 in circulation can bypass the blood-brain barrier into the brain tissue and cerebrospinal fluid, activating its receptors as GH does (31, 32). Previous studies have reported the GH-IGF-I axis plays an important role in multiple brain functions and might be targeted as a promising therapy for neurodegenerative diseases such as Amyotrophic lateral sclerosis, Alzheimer's disease, Parkinson's disease, and dementia (33–36). However, due to the pharmacological half-life of GH in the circulation and the cross-activation of GH/IGF-1 receptors, to distinguish the effects between GH and IGF-1 still remains difficult. As our previous case report has shown that GH could improve the outcomes and symptoms of PMS (16), this pilot study further investigated the efficacy and safety of rhGH and suggests that rhGH may improve the symptoms of PMS. Based on the results from GDS, gross motor function, fine motor function, language development, adaptive skills, and personal social behavior were significantly improved (*P* < 0.0083) following rhGH treatment; meanwhile, the results from SC-ABC showed that rhGH treatment also significantly improved sensory behavior, social relationships, body and object use, language and communication skills, and social and adaptive skills of the patients (*P* < 0.0083).

**Figure 2:**
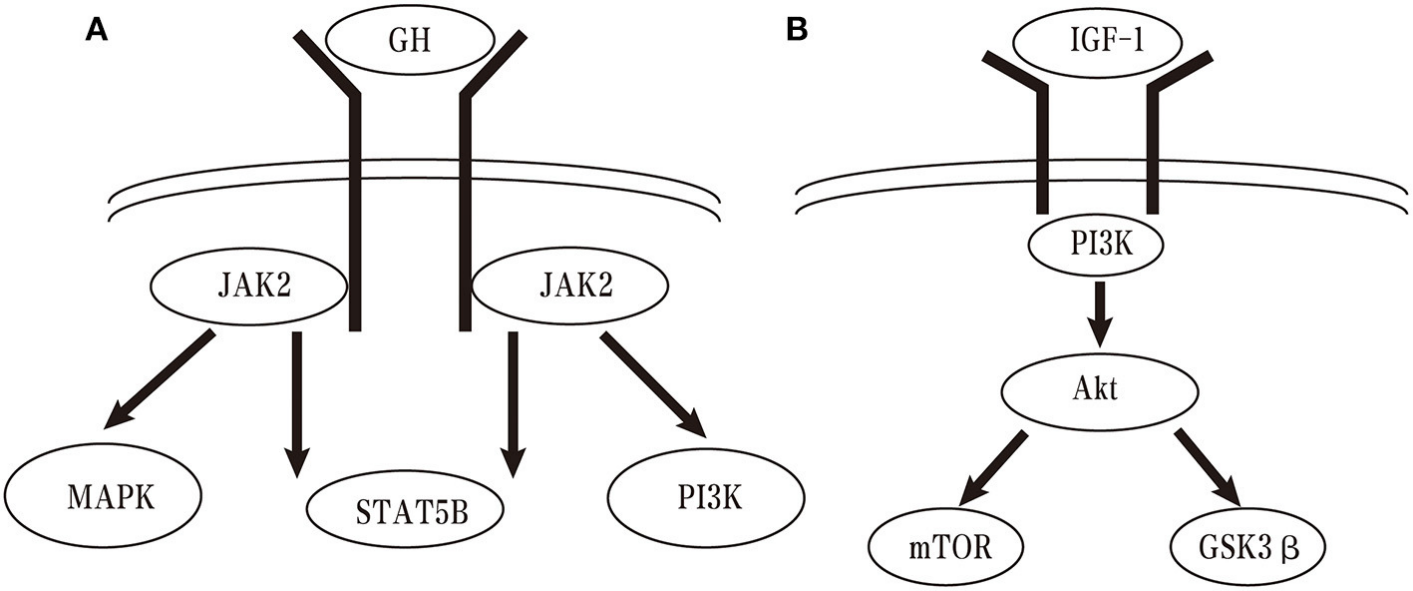
Schematic drawing of GH and IGF-1 signaling pathways.

The GH/IGF-I axis involves multiple different signaling pathways (Figure 2). GH promotes children growth mainly by regulating IGF-I production through growth hormone receptor (GHR). The classical GH-GHR signaling pathway involves the cytosolic Janus kinase 2 (JAK2) and signal transducer and activator of transcription 5B (STAT5B) (37). Several other pathways, such as mitogen-activated protein kinase (MAPK) and the phosphoinositide-3 kinase (PIK3) pathways, are also activated by the GHR-JAK2 system (38). JAK2 mutations have been proved to be the cause of several hematologic disorders such as myeloproliferative neoplasm, thrombocytopenia, and polycythemia Vera (39–41). STAT5B deficiency is tightly associated with growth hormone insensitivity (GHI) syndrome, IGF-I deficiency, and postnatal growth failure (42). MAPK is partially associated with GHI syndrome and PI3K is related to anti-apoptosis (43, 44). To date, however, it is still difficult to define the precise signaling of IGF-1 in neurodegenerative diseases (45, 46). The most investigated pathways of IGF-1 are PI3K/Akt/mammalian target of rapamycin (mTOR) and PI3K/Akt/GSK3β pathways. The IGF-1 is the most important pathway that promotes cell proliferation, growth, tumorigenesis and motility (47). Previous studies shown that more than 70% of human colorectal and breast cancer is linked to gene mutations or gene amplification altered by the PI3K/Akt/mTOR pathway (48, 49). Therefore, this pathway is an important pathway for targeted therapy and Food and Drug Administration (FDA) had already approved three PI3K inhibitors for cancer treatment. While the GSK3β, a downstream substrate of PI3K/Akt signaling induced by IGF-1, may involve the pathogenesis of Parkinson's Disease (PD) (50, 51). Hence, the PI3K/Akt/GSK3β signaling pathway may provide a new therapy for PD and further investigations are needed (52).

The specific mechanisms of GH in treating PMS are still unclear, which need further investigation. In the recent years, the GH-IGF-I axis signaling system has emerged as a target for developing novel new therapies for cancer (53, 54). Many GH-releasing hormone antagonists have been tested as anticancer therapies in preclinical studies due to their ability to inhibit the GH–IGF-I axis (55). The influence of GH on cancer risk in individuals without cancer history or cancer-related risk factors has been controversial for many years (54). A previous study demonstrated that GH cause no malignant transformation, while it can reduce the time length of DNA repair during the rapid progression of cell cycle, increasing the risk of gene mutation (53). In a long-term cohort study enrolling 6,928 subjects treated with GH in France, Carel et al. reported ~33% higher all-cause mortality in the subjects treated with GH, which was mainly attributed to the incidence of bone tumors and intracerebral hemorrhages (56). The clinical guidelines for children patients treated with GH were summarized from the Pediatric Endocrine Society (USA): (i) GH can be safely administered to children without inducing any known risk factor; (ii) in children with known predisposing conditions, GH therapy should be assessed on an individual basis and properly monitored (iii) for children who are cancer survivors in remission, GH can be administered with the understanding that it can enhance the risk of a second malignancy (57). The first clinical relationship between IGF1 and breast cancer was reported in 1959 (58). Previous systematic reviews and meta-analyses also suggested that the high level of IGF1 in circulation is associated with an increased risk of breast cancer (59). In 2020, a study investigated 206,263 women in the UK Biobank and revealed that high level IGF-1 in circulation could induce 40% increased risk for breast cancer in women older than 50 years old (60). However, further long- term observational studies are still needed to unravel the correlation between IGF-1 and cancer. In the present study, we detected a significant increase of serum IGF-1 and IGFBP-3 levels in the PMS patients, and we hypothesized that rhGH administration could improve PMS symptoms via increasing serum levels of IGF-1, and IGFBP-3.

## Limitations

This study has several limitations that need to be acknowledged: First, despite the considerable results obtained from this pilot study, the sample size was extremely small and participants' ages ranged from 1 to 5 years old, further studies in a larger sample size and/or investigating patients aged over 5 years old are needed. Second, we did not discuss the correlation between total scores difference of the measurements and deletion sizes in this study due to the sample size was extremely small. Third, a 4-week washout period was included to minimize the effects of rhGH on behavioral/developmental measurements based on the previous IGF-1 pilot study (15), bias on the measurements of SC-ABC and GDS still existed. In addition, since GH therapy requires properly monitored in children with known risk of cancer, further long-term studies involving cancer risk factors are needed to validate the safety of rhGH in treating PMS. Lastly, the influence of various dosage of rhGH was not included to ensure its safety in the patients.

## Conclusions

In summary, this pilot study involving six PMS children patients reveals that rhGH has a positive treatment effect on PMS. In addition, it is indicated that rhGH can improve PMS symptoms via increasing the level of serum IGF-1 and IGFBP-3 in the patients. These results encourage the undertaking of a large, randomized placebo-controlled trial to conclusively prove rhGH efficacy and tolerability in PMS, thereby promoting it as a low-cost, more accessible treatment for PMS, as compared to IGF-1.

## Data Availability Statement

The original contributions presented in the study are included in the article/Supplementary Material, further inquiries can be directed to the corresponding author/s.

## Ethics Statement

The studies involving human participants were reviewed and approved by Ethics Committee of Affiliated Hospital of Jiangnan University. Written informed consent to participate in this study was provided by the participants' legal guardian/next of kin.

## Author Contributions

TL and RX: design this study, collect and sort out data, and write papers. JZ: data analysis and revision of paper. HX, YC, and CW: provide clinical information and data. YL: reviewing and editing, final approval of the version to be published, funding acquisition. All authors contributed to the article and approved the submitted version.

## Funding

This study was supported by the Wuxi Key Medical Talents financial (No: ZDRC017).

## Conflict of Interest

The authors declare that the research was conducted in the absence of any commercial or financial relationships that could be construed as a potential conflict of interest.

## Publisher's Note

All claims expressed in this article are solely those of the authors and do not necessarily represent those of their affiliated organizations, or those of the publisher, the editors and the reviewers. Any product that may be evaluated in this article, or claim that may be made by its manufacturer, is not guaranteed or endorsed by the publisher.

## Supplementary Material

Supplementary File 1 Simplified Chinese version of the Aberrant Behavior Checklist.

Supplementary File 2 Detailed information of the semi-structured interview.

## References

[1] *PMS Cases Worldwide*,. (2021). Available online at: https://pmsf.org/about-pms/ (accessed January 17, 2022).

[2] Phelan K McDermid HE. The 22q13.3 deletion syndrome (Phelan-McDermid syndrome). Mol Syndromol. (2012) 2:186–201. 10.1159/000334260 22670140 PMC3366702

[3] Costales JL Kolevzon A. Phelan-McDermid syndrome and SHANK3: implications for treatment. Neurotherapeutics. (2015) 12:620–30. 10.1007/s13311-015-0352-z 25894671 PMC4489957

[4] Kolevzon A Angarita B Bush L Wang AT Frank Y Yang A. Phelan-McDermid syndrome: a review of the literature and practice parameters for medical assessment and monitoring. J Neurodev Disord. (2014) 6:39. 10.1186/1866-1955-6-39 25784960 PMC4362650

[5] Bonaglia MC Giorda R Beri S De Agostini C Novara F Fichera M. Molecular mechanisms generating and stabilizing terminal 22q13 deletions in 44 subjects with Phelan/McDermid syndrome. PLoS Genet. (2011) 7:e1002173. 10.1371/journal.pgen.1002173 21779178 PMC3136441

[6] Ricciardello A Tomaiuolo P Persico AM. Genotype-phenotype correlation in Phelan-McDermid syndrome: a comprehensive review of chromosome 22q13 deleted genes. Am J Med Genet A. (2021) 185:2211–33. 10.1002/ajmg.a.62222 33949759 PMC8251815

[7] Samogy-Costa CI Varella-Branco E Monfardini F Ferraz H Fock RA Barbosa RHA. A Brazilian cohort of individuals with Phelan-McDermid syndrome: genotype-phenotype correlation and identification of an atypical case. J Neurodev Disord. (2019) 11:13. 10.1186/s11689-019-9273-1 31319798 PMC6637483

[8] De Rubeis S Siper PM Durkin A Weissman J Muratet F Halpern D. Delineation of the genetic and clinical spectrum of Phelan-McDermid syndrome caused by SHANK3 point mutations. Mol Autism. (2018) 9:31. 10.1186/s13229-018-0205-9 29719671 PMC5921983

[9] Soorya L Kolevzon A Zweifach J Lim T Dobry Y Schwartz L. Prospective investigation of autism and genotype-phenotype correlations in 22q13 deletion syndrome and SHANK3 deficiency. Mol Autism. (2013) 4:18. 10.1186/2040-2392-4-18 23758760 PMC3707861

[10] Zhou Y Sharma J Ke Q Landman R Yuan J Chen H. Atypical behaviour and connectivity in SHANK3-mutant macaques. Nature. (2019) 570:326–31. 10.1038/s41586-019-1278-0 31189958

[11] Watt JL Olson IA Johnston AW Ross HS Couzin DA Stephen GS. A familial pericentric inversion of chromosome 22 with a recombinant subject illustrating a 'pure' partial monosomy syndrome. J Med Genet. (1985) 22:283–7. 10.1136/jmg.22.4.283 4045954 PMC1049449

[12] Shcheglovitov A Shcheglovitova O Yazawa M Portmann T Shu R Sebastiano V. SHANK3 and IGF1 restore synaptic deficits in neurons from 22q13 deletion syndrome patients. Nature. (2013) 503:267–71. 10.1038/nature12618 24132240 PMC5559273

[13] Manning MA Cassidy SB Clericuzio C Cherry AM Schwartz S Hudgins L. Terminal 22q deletion syndrome: a newly recognized cause of speech and language disability in the autism spectrum. Pediatrics. (2004) 114:451–7. 10.1542/peds.114.2.451 15286229

[14] Bozdagi O Tavassoli T Buxbaum JD. Insulin-like growth factor-1 rescues synaptic and motor deficits in a mouse model of autism and developmental delay. Mol Autism. (2013) 4:9. 10.1186/2040-2392-4-9 23621888 PMC3649942

[15] Kolevzon A Bush L Wang AT Halpern D Frank Y Grodberg D. A pilot controlled trial of insulin-like growth factor-1 in children with Phelan-McDermid syndrome. Mol Autism. (2014) 5:54. 10.1186/2040-2392-5-54 25685306 PMC4326443

[16] Xie RJ Li TX Sun C Cheng C Zhao J Xu H. A case report of Phelan-McDermid syndrome: preliminary results of the treatment with growth hormone therapy. Ital J Pediatr. (2021) 47:49. 10.1186/s13052-021-01003-w 33663540 PMC7934562

[17] Lord C Elsabbagh M Baird G Veenstra-Vanderweele J. Autism spectrum disorder. Lancet. (2018) 392:508–20. 10.1016/S0140-6736(18)31129-2 30078460 PMC7398158

[18] Liang Y. Guidelines of rhGH treatments in pedatrics. Chin J Pedatr. (2013) 51:426–32. 10.3760/cma.j.issn.0578-1310.2013.06.007

[19] Krug DA Arick J Almond P. behavior checklist for identifying severely handicapped individuals with high levels of autistic behavior. J Child Psychol Psychiatry. (1980) 21:221–9. 10.1111/j.1469-7610.1980.tb01797.x 7430288

[20] Karabekiroglu K Aman MG. Validity of the aberrant behavior checklist in a clinical sample of toddlers. Child Psychiatry Hum Dev. (2009) 40:99–110. 10.1007/s10578-008-0108-7 18600444

[21] Jun-Hong MA. Reliability and validity of the simplified chinese version of the aberrant behavior checklist in Beijing, China. Chin J Psychiatr. (2011) 25:14–9. 10.3969/j.issn.1000-6729.2011.01.00433173506

[22] Kat S Xu L Guo Y Ma J Ma Z Tang X. Reliability and validity of the simplified Chinese version of the aberrant behavior checklist in Chinese autism population. Front Psychiatry. (2020) 11:545445. 10.3389/fpsyt.2020.545445 33173506 PMC7591387

[23] De Sanctis V Soliman AT Candini G Yassin M Raiola G Galati MC. Insulin-like growth factor-1 (IGF-1): demographic, clinical and laboratory data in 120 consecutive adult patients with thalassaemia major. Mediterr J Hematol Infect Dis. (2014) 6:e2014074. 10.4084/mjhid.2014.074 25408860 PMC4235482

[24] Wang Y Zhang H Cao M Kong L Ge X. Analysis of the value and correlation of IGF-1 with GH and IGFBP-3 in the diagnosis of dwarfism. Exp Ther Med. (2019) 17:3689–93. 10.3892/etm.2019.7393 30988753 PMC6447816

[25] Yang A Choi JH Sohn YB Eom Y Lee J Yoo HW. Effects of recombinant human growth hormone treatment on growth, body composition, and safety in infants or toddlers with Prader-Willi syndrome: a randomized, active-controlled trial. Orphanet J Rare Dis. (2019) 14:216. 10.1186/s13023-019-1195-1 31511031 PMC6739953

[26] Armstrong RA. When to use the Bonferroni correction. Ophthalmic Physiol Opt. (2014) 34:502–8. 10.1111/opo.12131 24697967

[27] Francis G Thunell E. Reversing Bonferroni. Psychon Bull Rev. (2021) 28:788–94. 10.3758/s13423-020-01855-z 33464549

[28] Ranstam J. Hypothesis-generating and confirmatory studies, Bonferroni correction, and pre-specification of trial endpoints. Acta Orthop. (2019) 90:297. 10.1080/17453674.2019.1612624 31084234 PMC6718169

[29] Walser M Svensson J Karlsson L Motalleb R Åberg M Kuhn HG. Growth hormone and neuronal hemoglobin in the brain-roles in neuroprotection and neurodegenerative diseases. Front Endocrinol. (2020) 11:606089. 10.3389/fendo.2020.606089 33488521 PMC7821093

[30] Codrich M Bertuzzi M Russo R Francescatto M Espinoza S Zentilin L. Neuronal hemoglobin affects dopaminergic cells' response to stress. Cell Death Dis. (2017) 8:e2538. 10.1038/cddis.2016.458 28055011 PMC5386368

[31] Yuan T Ying J Jin L Li C Gui S Li Z. The role of serum growth hormone and insulin-like growth factor-1 in adult humans brain morphology. Aging. (2020) 12:1377–96. 10.18632/aging.102688 31967977 PMC7053622

[32] Martín-Rodríguez JF Ramos-Herrero VD Parras GG Flores-Martínez Á Madrazo-Atutxa A Cano DA. Chronic adult-onset of growth hormone/IGF-I hypersecretion improves cognitive functions and LTP and promotes neuronal differentiation in adult rats. Acta Physiol. (2020) 229:e13293. 10.1111/apha.13293 31059193

[33] Falleti MG Maruff P Burman P Harris A. The effects of growth hormone (GH) deficiency and GH replacement on cognitive performance in adults: a meta-analysis of the current literature. Psychoneuroendocrinology. (2006) 31:681–91. 10.1016/j.psyneuen.2006.01.005 16621325

[34] Scheepens A Williams CE Breier BH Guan J Gluckman PD. A role for the somatotropic axis in neural development, injury and disease. J Pediatr Endocrinol Metab. (2000) 13 (Suppl. 6):1483–91. 10.1515/jpem-2000-s623 11202225

[35] High WM Jr Briones-Galang M Clark JA Gilkison C Mossberg KA Zgaljardic DJ. Effect of growth hormone replacement therapy on cognition after traumatic brain injury. J Neurotrauma. (2010) 27:1565–75. 10.1089/neu.2009.1253 20578825 PMC2966848

[36] Bianchi VE Locatelli V Rizzi L. Neurotrophic and neuroregenerative effects of GH/IGF1. Int J Mol Sci. (2017) 18:2441. 10.3390/ijms18112441 29149058 PMC5713408

[37] Hwa V. Human growth disorders associated with impaired GH action: defects in STAT5B and JAK2. Mol Cell Endocrinol. (2021) 519:111063. 10.1016/j.mce.2020.111063 33122102 PMC7736371

[38] Brooks AJ Dai W O'Mara ML Abankwa D Chhabra Y Pelekanos RA. Mechanism of activation of protein kinase JAK2 by the growth hormone receptor. Science. (2014) 344:1249783. 10.1126/science.1249783 24833397

[39] Kralovics R Passamonti F Buser AS Teo SS Tiedt R Passweg JR. A gain-of-function mutation of JAK2 in myeloproliferative disorders. N Engl J Med. (2005) 352:1779–90. 10.1056/NEJMoa051113 15858187

[40] Baxter EJ Scott LM Campbell PJ East C Fourouclas N Swanton S. Acquired mutation of the tyrosine kinase JAK2 in human myeloproliferative disorders. Lancet. (2005) 365:1054–61. 10.1016/S0140-6736(05)71142-9 15781101

[41] Tefferi A Lasho TL Gilliland G. JAK2 mutations in myeloproliferative disorders. N Engl J Med. (2005) 353:1416–7. 10.1056/NEJMc051878 16192494

[42] Hwa V Nadeau K Wit JM Rosenfeld RG. STAT5b deficiency: lessons from STAT5b gene mutations. Best Pract Res Clin Endocrinol Metab. (2011) 25:61–75. 10.1016/j.beem.2010.09.003 21396575

[43] Gong Y Luo S Fan P Zhu H Li Y Huang W. Growth hormone activates PI3K/Akt signaling and inhibits ROS accumulation and apoptosis in granulosa cells of patients with polycystic ovary syndrome. Reprod Biol Endocrinol. (2020) 18:121. 10.1186/s12958-020-00677-x 33287836 PMC7720521

[44] Malaquias AC Jorge AAL. Activation of the MAPK pathway (RASopathies) and partial growth hormone insensitivity. Mol Cell Endocrinol. (2021) 519:111040. 10.1016/j.mce.2020.111040 33011209

[45] Johnson SC. Nutrient sensing, signaling and ageing: the role of IGF-1 and mTOR in ageing and age-related disease. Subcell Biochem. (2018) 90:49–97. 10.1007/978-981-13-2835-0_3 30779006

[46] Costales J Kolevzon A. The therapeutic potential of insulin-like growth factor-1 in central nervous system disorders. Neurosci Biobehav Rev. (2016) 63:207–22. 10.1016/j.neubiorev.2016.01.001 26780584 PMC4790729

[47] Alzahrani AS. PI3K/Akt/mTOR inhibitors in cancer: at the bench and bedside. Semin Cancer Biol. (2019) 59:125–32. 10.1016/j.semcancer.2019.07.009 31323288

[48] Miricescu D Totan A Stanescu S II Badoiu SC Stefani C Greabu M. PI3K/AKT/mTOR signaling pathway in breast cancer: from molecular landscape to clinical aspects. Int J Mol Sci. (2020) 22:173. 10.3390/ijms22010173 33375317 PMC7796017

[49] Hao Y Samuels Y Li Q Krokowski D Guan BJ Wang C. Oncogenic PIK3CA mutations reprogram glutamine metabolism in colorectal cancer. Nat Commun. (2016) 7:11971. 10.1038/ncomms11971 27321283 PMC4915131

[50] Wang Y Liu W He X Zhou F. Parkinson's disease-associated DJ-1 mutations increase abnormal phosphorylation of tau protein through Akt/GSK-3β pathways. J Mol Neurosci. (2013) 51:911–8. 10.1007/s12031-013-0099-0 23979838

[51] Offen D Shtaif B Hadad D Weizman A Melamed E Gil-Ad I. Protective effect of insulin-like-growth-factor-1 against dopamine-induced neurotoxicity in human and rodent neuronal cultures: possible implications for Parkinson's disease. Neurosci Lett. (2001) 316:129–32. 10.1016/S0304-3940(01)02344-8 11744219

[52] Yang L Wang H Liu L Xie A. The role of insulin/IGF-1/PI3K/Akt/GSK3β signaling in Parkinson's disease dementia. Front Neurosci. (2018) 12:73. 10.3389/fnins.2018.00073 29515352 PMC5826217

[53] Werner H Laron Z. Role of the GH-IGF1 system in progression of cancer. Mol Cell Endocrinol. (2020) 518:111003. 10.1016/j.mce.2020.111003 32919021

[54] Boguszewski CL Boguszewski M. Growth hormone's links to cancer. Endocr Rev. (2019) 40:558–574. 10.1210/er.2018-00166 30500870

[55] Zarandi M Cai R Kovacs M Popovics P Szalontay L Cui T. Synthesis and structure-activity studies on novel analogs of human growth hormone releasing hormone (GHRH) with enhanced inhibitory activities on tumor growth. Peptides. (2017) 89:60–70. 10.1016/j.peptides.2017.01.009 28130121

[56] Carel JC Ecosse E Landier F Meguellati-Hakkas D Kaguelidou F Rey G. Long-term mortality after recombinant growth hormone treatment for isolated growth hormone deficiency or childhood short stature: preliminary report of the French SAGhE study. J Clin Endocrinol Metab. (2012) 97:416–25. 10.1210/jc.2011-1995 22238382

[57] Raman S Grimberg A Waguespack SG Miller BS Sklar CA Meacham LR. Risk of neoplasia in pediatric patients receiving growth hormone therapy–a report from the pediatric endocrine society drug and therapeutics committee. J Clin Endocrinol Metab. (2015) 100:2192–203. 10.1210/jc.2015-1002 25839904 PMC5393518

[58] Pearson OH Ray BS. Results of hypophysectomy in the treatment of metastatic mammary carcinoma. Cancer. (1959) 12:85–92. 10.1002/1097-0142(195901/02)12:1<85::AID-CNCR2820120114>3.0.CO 13618860

[59] Sklar CA. Growth hormone treatment: cancer risk. Horm Res. (2004) 62 (Suppl. 3):30–4. 10.1159/000080496 15539796

[60] Murphy N Knuppel A Papadimitriou N Martin RM Tsilidis KK Smith-Byrne K. Insulin-like growth factor-1, insulin-like growth factor-binding protein-3, and breast cancer risk: observational and Mendelian randomization analyses with ~430 000 women. Ann Oncol. (2020) 31:641–9. 10.1016/j.annonc.2020.01.066 32169310 PMC7221341

