# Supplementary material for: Effectiveness of Recombinant Human Growth Hormone Therapy for Children With Phelan-McDermid Syndrome: An Open-Label, Cross-Over, Preliminary Study
Source: Front Psychiatry. 2022 Feb 16;13:763565. doi: 10.3389/fpsyt.2022.763565 (PMC8888442; doi:10.3389/fpsyt.2022.763565)
Supplement: Supplementary File 1 — Simplified Chinese version of the Aberrant Behavior Checklist. [file Table_1.DOCX]

**Simplified Chinese version of the Aberrant Behavior Checklist (SC-ABC)**

The scale is the most common autism screening scale used in China, with 57 items, including 5 factors: sensory, relating, body and object use, language and social and self-help. Each item is scored 0-3 by the caregiver according to the patient's behaviors in the past 1 month.

Circle the number for those items most accurately describing the child:

| **Projects** | | **Answer** | | **Score** |
| --- | --- | --- | --- | --- |
|  |  | **yes** | **no** |  |
| 1 | Whirls self for long periods of time |  |  |  |
| 2 | Learns a simple task but "forgets" quickly |  |  |  |
| 3 | Frequently does not attend to social/environmental cues |  |  |  |
| 4 | Does not follow simple commands (sit down, come here, stand up) given once |  |  |  |
| 5 | Does not use toys appropriately (spins wheels, etc) |  |  |  |
| 6 | Poor use of visual discrimination when learning (fixates on parts of objects such as size, color, position... |  |  |  |
| 7 | Lacks a social smile (may smile out-of-context) |  |  |  |
| 8 | Exhibits pronoun reversal (you for I...) |  |  |  |
| 9 | Insists on keeping certain objects with him/herself |  |  |  |
| 10 | Seems not to hear (despite normal hearing tests) |  |  |  |
| 11 | Speech is atonal and arrhythmic |  |  |  |
| 12 | Rocks self for long periods of time |  |  |  |
| 13 | Does not (or did not as a baby) reach out when reached for |  |  |  |
| 14 | Strong reactions to minor changes in routine/environment |  |  |  |
| 15 | Does not respond to own name when called out among two or more other names |  |  |  |
| 16 | Lunges and darts about, interrupted by spinning, toe walking, hand flapping... |  |  |  |
| 17 | Not responsive to other people's facial expressions or feeings |  |  |  |
| 18 | Seldom uses "yes"or‘I" |  |  |  |
| 19 | Has special abilities in one area - seems to rule out mental retardation |  |  |  |
| 20 | Does not follow simple prepositional commands (e.g., "put the ball in the box") |  |  |  |
| 21 | Sometimes shows no"startle response" to a loud noise |  |  |  |
| 22 | Flaps hands (or other self-simulating behavior) |  |  |  |
| 23 | Severe temper tantrums and/or frequent minor tantrums |  |  |  |
| 24 | Actively avoids eye contact |  |  |  |
| 25 | Resists being touched or held |  |  |  |
| 26 | Sometimes, painful stimuli (cuts, injections, bruises) evoke no reaction |  |  |  |
| 27 | Is (or was as a baby) stiff and hard to hold |  |  |  |
| 28 | Is flaccid (doesn't cling) when held in arms |  |  |  |
| 29 | Gets desired objects by gesturing |  |  |  |
| 30 | Walks on toes |  |  |  |
| 31 | Huts others by biting, hitting, kicking... |  |  |  |
| 32 | Repeats phrases over and over again |  |  |  |
| 33 | Does not imitate other children at play |  |  |  |
| 34 | Often will not blink when a bright light is directed toward eyes |  |  |  |
| 35 | Hurts self by biting hand, banging head... |  |  |  |
| 36 | Does not wait for needs to be met (wants things immediately) |  |  |  |
| 37 | Cannot point to more than five named objects |  |  |  |
| 38 | Has not developed any friendships |  |  |  |
| 39 | Covers ears at many sounds |  |  |  |
| 40 | Twirls, spins, and bangs objects a lot |  |  |  |
| 41 | Difficulties with toilet training |  |  |  |
| 42 | Uses 5 or less words per day spontaneously to communicate wants or needs |  |  |  |
| 43 | Often frightened or very anxious |  |  |  |
| 44 | Squints, frowns, or covers eyes when in the presence of natural light |  |  |  |
| 45 | Does not dress self without frequent help |  |  |  |
| 46 | Repeats sounds or word over and over again |  |  |  |
| 47 | "Looks through"people |  |  |  |
| 48 | Echoes questions or statements made by other people |  |  |  |
| 49 | Frequently unaware of surroundings and may be oblivious to dangerous situations |  |  |  |
| 50 | Prefers to manipulate and be occupied with inanimate objects |  |  |  |
| 51 | Will feel, smell, or taste objects in the environment |  |  |  |
| 52 | Frequently has no visual reaction to a "new" person |  |  |  |
| 53 | Gets involved in complicated "rituals" such as lining things up... |  |  |  |
| 54 | Is very destructive (toys and household items are quickly broken) |  |  |  |
| 55 | A developmental delay was identified at or before 30 months of age |  |  |  |
| 56 | Uses at least 15 but less than 30 spontaneous phrases daily to communicate |  |  |  |
| 57 | Stares into space for long periods of time |  |  |  |
| Total |  |  | | |
