# Supplementary material for: Effectiveness of Recombinant Human Growth Hormone Therapy for Children With Phelan-McDermid Syndrome: An Open-Label, Cross-Over, Preliminary Study
Source: Front Psychiatry. 2022 Feb 16;13:763565. doi: 10.3389/fpsyt.2022.763565 (PMC8888442; doi:10.3389/fpsyt.2022.763565)
Supplement: Supplementary File 2 — Detailed information of the semi-structured interview. [file Table_2.DOCX]

**Semi-structured interview about adverse events**

The interview including 4 parts: common adverse events, serious adverse events, local reactions at injection site, and other adverse events. Each item documented for severity, duration, and management in the past two weeks.

| *Common adverse events* | Severity | Duration | Management |
| --- | --- | --- | --- |
| 1. Upper respiratory tract infection |  |  |  |
| 2. Nasopharyngitis |  |  |  |
| 3. Constipation |  |  |  |
| 4. Pyrexia |  |  |  |
| 5. Pneumonia |  |  |  |
| 6. Bronchitis |  |  |  |
| *Serious adverse events* |  |  |  |
| 1. Infections and infestations^1^ |  |  |  |
| 2. Nervous system disorders^2^ |  |  |  |
| 3. Enteritis |  |  |  |
| 4. Congestive cardiomyopathy |  |  |  |
| 5. Strabismus |  |  |  |
| 6. Middle ear effusion |  |  |  |
| *Local reactions at injection site* |  |  |  |
| 1. Warmth |  |  |  |
| 2. Erythema |  |  |  |
| 3. Swelling |  |  |  |
| *Other adverse events* |  |  |  |
| *Please provide the symptoms and other information* |  |  |  |
|  |  |  |  |
|  |  |  |  |
|  |  |  |  |

^1^Infections and infestations: including bronchiolitis, bronchitis, pneumonia, upper respiratory tract infection, urinary tract infection and viral infection.

^2^ Nervous system disorders: including febrile convulsion and seizure
